# Electrochemical Sensor Based on the Hierarchical Carbon Nanocomposite for Highly Sensitive Ciprofloxacin Determination

**DOI:** 10.3390/membranes13070682

**Published:** 2023-07-21

**Authors:** Joanna Smajdor, Beata Paczosa-Bator, Robert Piech

**Affiliations:** Department of Analytical Chemistry and Biochemistry, Faculty of Materials Science and Ceramics, AGH University of Science and Technology, Al. Mickiewicza, 30-059 Krakow, Poland

**Keywords:** ciprofloxacin, voltammetry, nanocomposite, carbon nanomaterials

## Abstract

A new voltammetry method for the highly sensitive antibacterial drug ciprofloxacin (CIP) is presented using glassy carbon electrodes modified with hierarchical electrospun carbon nanofibers with NiCo nanoparticles (eCNF/CNT/NiCo-GCE). The use of a modified glassy carbon electrode in the form of hierarchical electrospun carbon nanofibers with NiCo nanoparticles (eCNF/CNT/NiCo) led to an LOD value as low as 6.0 µmol L^−1^ with a measurement sensitivity of 3.33 µA µmol L^−1^. The described procedure was successfully applied for CIP determination in samples with complex matrices, such as urine or plasma, and also in pharmaceutical products and antibiotic discs with satisfactory recovery values ranging between 94–104%. The proposed electrode was characterised by great stability, with the possibility of use for about 4 weeks without any significant change in the CIP peak current. The repeatability of the CIP response on the eCNF/CNT/NiCo/GC is also very good; its value measured and expressed as RSD is equal to 2.4% for a CIP concentration of 0.025 µmol L^−1^ (for 7 consecutive CIP voltammogram registrations). The procedure for electrode preparation is quick and simple and does not involve the use of expensive apparatus.

## 1. Introduction

Ciprofloxacin (CIP) is an organic chemical compound and a chemotherapeutic agent of the group of second-generation quinolones (fluoroquinolones) with bactericidal activity, showing its effect by inhibiting bacterial DNA topoisomerase and DNA gyrase [1]. It is one of the most potent drugs among fluoroquinolones. The excreted material is mainly unchanged in the urine and partly in the feces, reaching high concentrations there [2]. It can be taken by mouth, as eye drops, as ear drops, or intravenously.

Ciprofloxacin is particularly potent against Gram-negative bacteria, such as *Escherichia coli*, *Haemophilus influenzae*, *Klebsiella pneumoniae*, *Legionella pneumophila*, *Moraxella catarrhalis*, *Proteus mirabilis*, and *Pseudomonas aeruginosa*, and less effective against Gram-positive bacteria, such as methicillin-sensitive *Staphylococcus aureus*, *Streptococcus pneumoniae*, and *Enterococcus faecalis* than newer generations of fluoroquinolones [1,3]. It is used mainly to treat urinary tract infections. Due to the fact that it penetrates well into the lungs, prostate gland, bones, cerebrospinal fluid, and palatine tonsils, it is also commonly used to treat infections of these organs. It is also effective in treating typhoid fever and anthrax infections [4,5].

Various analytical methods have been reported in the literature for determining CIP. The most popular involve spectrophotometric methods [6,7,8,9,10], fluorimetry [11,12], capillary electrophoresis [13,14,15], and high-performance liquid chromatography coupled with different detectors [16,17,18,19,20]. However, the methods mentioned require well-trained analysts to perform the analysis, expensive equipment, and a relatively long time for a single analysis. All those inconveniences led to the elaboration of the same precise and sensitive methods mentioned above, but with lower financial outlays and staff requirements. Therefore, the electrochemical methods gained scientists’ attention, meeting all of the above criteria. Not only are they characterised by the high sensitivity of measurements, but they can also help get information about the mechanisms of reactions or the kinetics of electron transfer and let you conduct the measurements in different kinds of matrices. A variety of working electrodes have been used for the voltametric determination of ciprofloxacin, including mercury electrodes [21,22], boron-doped diamond electrodes (BDD) [23], screen-printed electrodes (SPE) [24,25,26], carbon paste electrodes (CPE) [27,28,29] and glassy carbon electrodes modified with different nanomaterials, such as carbon nanotubes or metal oxides [30,31,32,33,34].

The field of carbon nanomaterials is firmly developing nowadays in the sensor construction area. The most popular in electrochemical applications include fullerenes, graphene, carbon nanotubes (CNT) and nanofibers (CNF), nanorods, carbon black (CB), etc., and each of them may be characterised by slightly different properties. In this work, the hierarchical composite consisting of CNTs and CNFs was used because of the favourable resultant properties of their properties. CNTs are a type of tube made of carbon with a diameter measured in nanometers [35,36]. They might be obtained in the production process with different morphologies, which influence their final properties. Many methods of CNT preparation have been reported in the literature so far, such as arc discharge, chemical vapour deposition (CVD), laser ablation, catalyst chemical vapour deposition (CCVD), and temperature-directed synthesis [37,38,39,40]. Carbon nanofibers are interesting compounds for the design of composites for analytical use. CNFs are carbon nanostructures with a cylindrical shape consisting of graphene layers arranged as stacked cones, cups, or plates. Carbon nanofibers are often synthesised using the catalytic decomposition of carbon-containing gases in the presence of metal particles [41,42]. The final product of such synthesis usually contains the leftover metal nanoparticles left after the preparation process. In some cases, such contamination might be advantageous, especially for the electrochemical application of such material [43,44]. Both types of carbon nanomaterials are known to occur in a wide variety of structural morphologies with different properties, considering internal order, texture, or surface development.

The aim of the presented study was to propose a new procedure for CIP determination using the glassy carbon electrode modified with the hierarchical nanocomposite: electrospun carbon nanofibers/carbon nanotubes/NiCo nanoparticles (eCNF/CNT/NiCo-GCe). The proposed electrode, together with the proposed measurement testing, guarantee high sensitivity of the measurement, short analysis time, significant sensor stability, and reproducibility of the results. The described method was successfully applied for the determination of CIP in complex matrices such as plasma and urine, and it is also suitable for its measurements in pharmaceutical and diagnostic products, such as popular antibiotics and antibiotic discs used for bacterial resistance tests.

## 2. Materials and Methods

### 2.1. Measuring Apparatus

For all voltametric measurements, an mtm-ANKO electrochemical analyzer, type 161, with the electrode stand and dedicated software, was used. All experiments were carried out in the classical three-electrode voltametric cell with a volume of 10 mL. As a working electrode, a glassy carbon electrode coated with the modifier layer consisted of hierarchical nanocomposite—electrospun carbon nanofibers/carbon nanotubes/NiCo nanoparticles (eCNF/CNT/NiCo-GCe) was chosen; also, a double junction reference electrode Ag/AgCl/KCl (3 mol L^−1^) and the platinum wire as an auxiliary electrode were part of the electrochemical cell. A magnetic bar with a rotating speed of 500 rpm was used in the preconcentration step. pH measurements were performed using a multipurpose laboratory instrument, the Elmetron CX-705 (Zabrze, Poland). Sonication of the prepared solutions was carried out using an ultrasonic bath (Intersonic IS-1K, Olsztyn, Poland). The modifier suspension was prepared according to the procedure described in the work [35,36]. The working electrode has been prepared according to the following procedures:

1. thermal treatment of non-woven carbon in a tubular furnace;
2. fragmentation of the obtained composite in a mortar;
3. mixing the powder with the DMF to obtain a suspension with a concentration of 1 mg mL^−1^;
4. homogenization of the suspension on the ultrasonic washer (Intersonic, IS-1K, Olsztyn, Poland) for 15 min;
5. preparation of the GC electrode surface by polishing with an alumina slurry with a particle size of 0.3 µm (Buehler Micropolish II, Chicago, IL, USA);
6. rinsing the leftovers of the alumina slurry with double-distilled water and methanol;
7. application of 7.5 µL of modifier suspension to the surface of the GC electrode surface with an automatic pipette (Eppendorf, Hamburg, Germany)
8. drying electrodes for about 12 h at room temperature.

### 2.2. Chemicals

Ciprofloxacin hydrochloride (CIP) was purchased from Sigma-Aldrich. The standard solution of ciprofloxacin (0.01 mol L^−^^1^, 5 mL) was obtained by dissolving the proper amount of analyte in water and hydrochloric acid (Merck) (4:1). Phosphate buffer (1 mol L^−^^1^, pH 6.8) was prepared by mixing the proper amounts of KH_2_PO_4_ and K_2_HPO_4_ (both Merck). Freeze-dried urine (Medidrug^®^ Basis-line U) was purchased from Medichem (Barcelona, Spain), and human plasma was purchased from Biowest (Nuaillé, France). All reagents were of analytical grade and used without further purification. All solutions were prepared with double-distilled water.

### 2.3. Sample Preparation

#### 2.3.1. Pharmaceutical Samples Preparation

CIP measurements in pharmaceutical formulations were performed on two types of antibiotic drugs, both containing 500 mg of ciprofloxacin hydrochloride, purchased in the local drugstore. For the quantitative analysis of ciprofloxacin in the pharmaceutical formulation, three of them were crushed in a mortar, and then a properly measured weight was quantitatively transferred to the volumetric flask and dissolved in double-distilled water and hydrochloric acid (properties 4:1) to obtain the desired concentration. The solution obtained was sonicated for 5 min to obtain homogenization throughout the volume. After this preparation, the solution was filtrated using syringe cellulose filters (pore size 0.45 µm) (Biosens, Warsaw, Poland) and used for further measurements.

#### 2.3.2. Antibiotic Disc

Ciprofloxacin content was measured in the antibiotic disc containing 5 μg of CIP (Oxoid, UK). The disc was added to the measurement cell, including 10 mL of support electrolyte, without prior preparation.

#### 2.3.3. Urine Preparation

Commercially frozen, dried urine samples were purchased by Medidrug (Barcelona, Spain) and prepared according to manufacturer procedures. The contents of the bottles were completely dissolved with double-distilled water and filtered with a RC syringe filter with a pore size of 0.45 m (Biosens). After this preparation, the sample was used for CIP measurements.

#### 2.3.4. Plasma Preparation

A commercial plasma sample was purchased from Biowest (France) and stored in the freezer at −20 °C. To remove potentially interfering protein components from the sample, 800 μL of plasma was mixed with 200 μL of 10% trichloroacetic acid (TCA), shaken for 2 min on the vortex (Biosan), and then centrifuged for 30 min at an approximate speed of 10,000 rpm (Eppendorf). The supernatant was filtered through the syringe filters with the RC membrane and a pore size of 0.45 μm (Biosens) and used for the CIP measurements.

### 2.4. Standard Procedure of Measurements

Quantitative analysis of ciprofloxacin was performed using the differential pulse voltammetry (DPV) technique. Measurements were carried out in the pH 6.8 0.1 mol L^−^^1^ phosphate buffer as a supporting electrolyte. The instrumental parameters of the highly sensitive CIP measurements were as follows: sampling and waiting time t_p_ = t_w_ = 10 ms, step potential E_s_ = 5 mV, and pulse amplitude dE = 50 mV. The voltammograms were registered in the potential range of 300 to 1100 mV after the preconcentration step described by the parameters E_acc_ = 300 mV and t_acc_ = 10 s. Between each voltammogram registration, a rest period of 20 s was strictly required to obtain the high repeatability of the CIP signals.

## 3. Results

### 3.1. Influence of the Volume of eCNF/CNT/NiCo Layer on the Glassy Carbon Electrode on the Ciprofloxacin Peak

**Figure 1:**
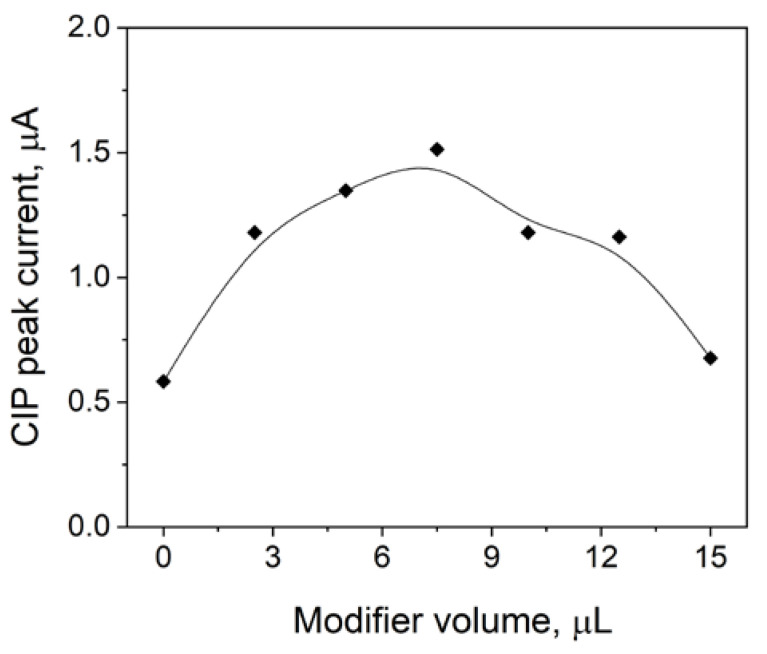
Relation between 0.5 µmol L^−1^ CIP peak current and volume of modifier used in the measurement. Instrumental parameters were as follows: E_s_ = 5 mV, dE = 50 mV, t_p_ = t_w_ = 10 ms, t_acc_ = 10 s, E_acc_ = 300 mV.

In this work, a modifier in the form of hierarchical electrospun carbon nanofibers with NiCo nanoparticles (eCNF/CNT/NiCo) was used to increase the sensitivity of the determination of the proposed method of ciprofloxacin [44,45]. The choice of modifier layer thickness was an important step during the optimization process of the proposed method. Too thick of a film may negatively influence the process of gathering the analyte on the electrode’s surface; therefore, the registered current will be lower. The key was to choose the volume of eCNF/CNT/NiCo that significantly developed the GC surface and consequently increased the CIP current response in comparison with the bare electrode, which was the crucial aim of this study. The measurements show that the CIP response on such a proposed electrode was strongly dependent on the amount of modifier applied to it (Figure 1). The use of the modifier strengthens the CIP peak current three times compared to the non-modified electrode (for a CIP concentration of 0.5 µmol L^−1^). Due to the application of the eCNF/CNT/NiCo layer on the GCE, the measured sensitivity and CIP limits of detection could be significantly better than those on the GC electrode. The optimal volume of the eCNF/CNT/NiCo layer in the GCE was set at 7.5 µL and used in all further studies.

### 3.2. Influence of the Supporting Electrolyte Composition on the Ciprofloxacin Peak

The influence of different types of supporting electrolytes on the height and shape of the ciprofloxacin peak was examined. In this case, the following solutions were tested: 0.1 mol L^−1^ potassium dihydrogen phosphate (pH 4.6), 0.1 mol L^−1^ dipotassium phosphate (pH 9.1), 0.1 mol L^−^^1^ phosphate buffers with pH values from 5.5 to 8.0; 0.5 mol L^−^^1^ acetate buffers with pH values 3.8, 4.5 and 5.5; 0.1 mol L^−^^1^ sodium tetraborate solution pH 10; 0.5 mol L^−^^1^ ammonia buffer pH 8.2 and 0.1× phosphate-buffered saline (pH 7.4) (Figure not included). Based on the results obtained, it was possible to say that the CIP peak represents the best properties, considering the peak current value, repeatability, and its shape in the 0.1 mol L^−^^1^ phosphate buffer with a pH of 6.8. Therefore, this solution was applied to further measurements.

### 3.3. Voltammetric Behaviour of Ciprofloxacin on eCNF/CNT/NiCo/GC Electrode

**Figure 2:**
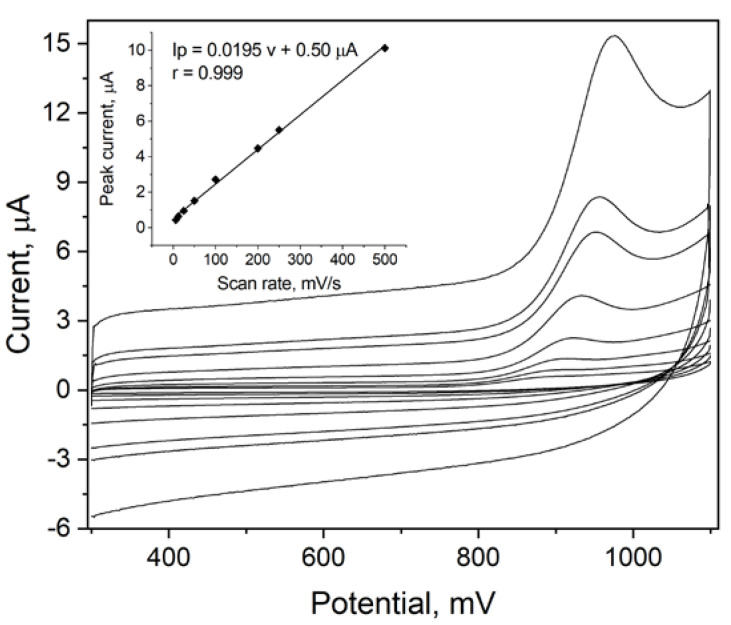
Cyclic voltammograms of 1 µmol L^−1^ ciprofloxacin measured in a 0.1 mol L^−1^ phosphate buffer (pH 6.8) on the eCNF/CNT/NiCo/GC electrode. The scan rate values were as follows: 6.3; 12.5; 25; 50; 100; 200; 250; and 500 mV s^−1^.

The behaviour of CIP on the eCNF/CNT/NiCo/GC was investigated using Cyclic Voltammetry. The scan rate values changed between 6.3 and 500 mV s^−1^ and measurements were performed in the supporting electrolyte consisting of 0.1 mol L^−1^ phosphate buffer pH 6.8 (Figure 2). It was observed that the CIP peak was shifting towards higher potential values with increasing scan rates. The linear dependence between the peak current and the scan rate was obtained; therefore, it is possible to say that the CIP oxidation process is controlled by adsorption. Moreover, considering the analysis of the CV results, the number of electrons taking part in the oxidation process was calculated [46]. In this respect, the plot of the dependence between the CIP peak potential and the natural logarithm of the scan rate was crossed out. Taking into account the slope value, the number of electrons calculated was equal to two. Taking into account the irreversible process of oxidation of CIP on the proposed electrode, the number of electrons exchanged can be calculated from CV curves knowing the values of the peak and half-peak potential [47]. The difference between these values might be expressed with the simple equation [47,48]:

Ep−Ep2=−1.857RTαnαF

Which, in the conditions of 20 °C, takes the form of:

αn=0.048Ep−Ep12

**Scheme 1:**
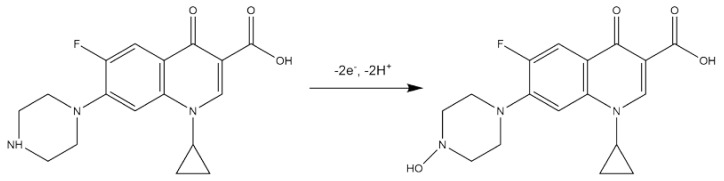
Possible scheme of ciprofloxacin oxidation on the eCNF/CNT/NiCo/GC electrode surface.

Therefore, the electron calculation using the following equation has been performed. Taking into account the peak and half-peak potential values and the alpha coefficient equal to 0.5 for irreversible processes, the result obtained was in great agreement with previous calculations, equal to 2. The scheme of possible CIP oxidation processes on the surface of eCNF/CNT/NiCo/GC electrodes is presented in Scheme 1.

**Figure 3:**
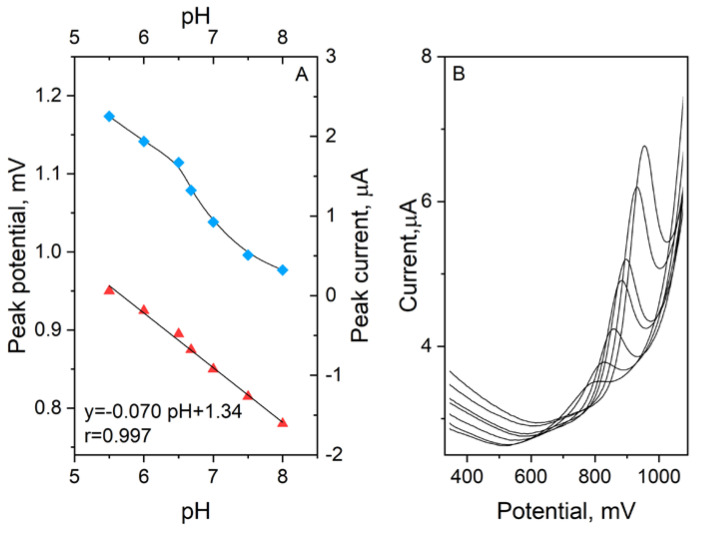
Plots of the ciprofloxacin peak current (blue) and potential (red) dependence on the supporting electrolyte pH in the range 5.5–8.0 (**A**) and corresponding DPV voltammograms of 0.5 µmol L^−1^ ciprofloxacin for pH in the range 5.5–8.0 measured in a 0.1 mol L^−1^ phosphate buffer (**B**). Instrumental parameters as in point 2.4.

In addition, the CIP behaviour of the supporting electrolyte, which consisted of phosphate buffer with different pH values, was investigated. In this case, a series of 0.1 mol L^−^^1^ phosphate buffers were prepared with pH values in the range from 5.5 to 8.0 and the 0.5 µmol L^−1^ CIP peak of 0.5 mol L^−^^1^ was registered in each of them. The results of the measurements, consisting of both the CIP peak current and the potential dependence on supporting electrolyte pH, along with the corresponding voltammograms, are presented in Figure 3. The linear equation of the CIP peak potential based on the pH value was obtained in the form of:

$$E_{p}=-0.070pH+1.34V$$

It is visible that the CIP peak signal was shifting towards lower potential values with increasing supporting electrolyte buffer pH values. Therefore, the increase in the pH value results in increasing the speed of electron transfer on the surface of the eCNF/CNT/NiCo/GC electrode, which influences the velocity of the whole CIP oxidation reaction. Taking into account the results obtained, the 0.1 mol L^−^^1^ phosphate buffer with a pH of 6.8 was chosen considering the most preferable properties, such as a high peak current value and a good repeatability of the signal.

### 3.4. Preconcentration Time and Potential Influence on the Ciprofloxacin Peak

**Figure 4:**
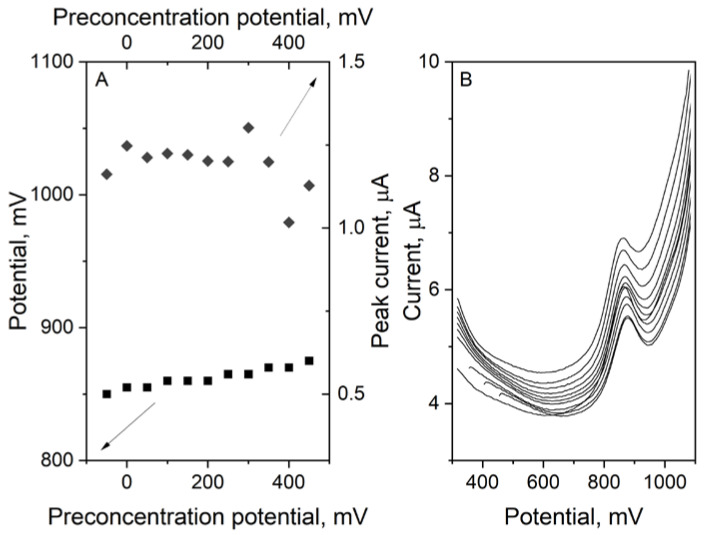
The influence of the preconcentration potential in the range from −50 mV to 450 mV on CIP peak current (rhombus) and potential (squares) (**A**) and corresponding voltammograms (**B**). Instrumental parameters as in point 2.4.

The key to obtaining the lowest possible detection limit is to optimise the DPV measurement process. One of the most crucial parameters that condition the LOD is the preconcentration potential (p_acc_) and the time (t_acc_), which may strongly influence the height of the analyte signal. The lengthening of the preconcentration time results in a higher CIP peak because the compound has more time to get to the electrode surface from further places in the supporting electrolyte. The amount of analyte accumulated on the surface of the modified electrode depends on the size of its active surface. The use of nanomaterials as electrode modifiers enlarges their surface because of their high surface development, which increases the available space for analyte accumulation. Therefore, the necessary step in the optimalization process is to choose the best values for both parameters. In this case, both t_acc_ and p_acc_ were investigated for a wide range of values. The influence of the accumulation potential was tested from a value of −50 mV to a value of 450 mV (Figure 4). The CIP peak was shifted towards higher potential values along with the growing value of the preconcentration potential, reaching values of 850 mV for the p_acc_ of −50 mV and 875 mV for the p_acc_ of 450 mV. Furthermore, the CIP peak current was changing with the change of the p_acc_, reaching the highest values for the p_acc_ of 300 mV and the lowest for 400 mV (a change of 24%). Therefore, the value of 300 mV was chosen as the best condition for high-sensitivity CIP determination.

**Figure 5:**
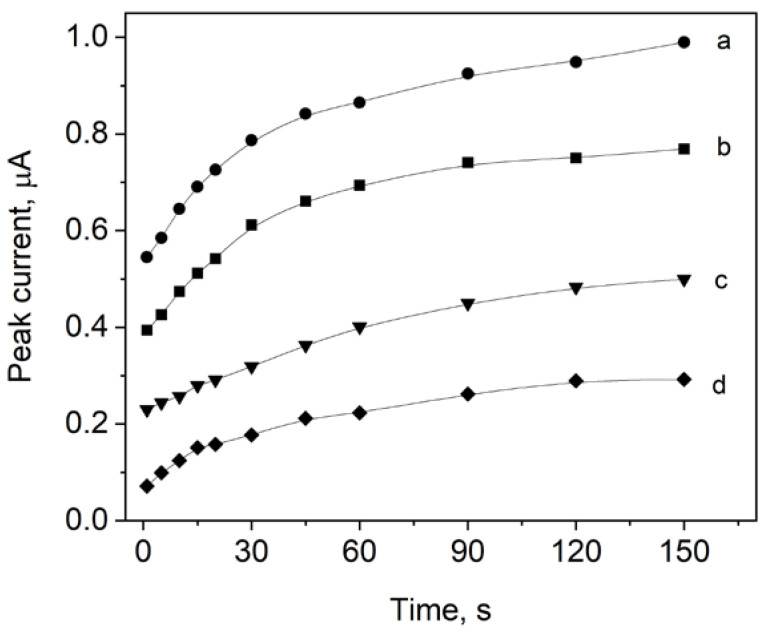
Dependence of the ciprofloxacin peak current on the value of preconcentration time. CIP concentrations are as follows: (a) 0.5; (b) 0.25; (c) 0.01 and (d) 0.005 μmol L^−^^1^. Other instrumental parameters as in point 2.4.

The influence of accumulation time was investigated for four different concentrations of ciprofloxacin: 0.5; 0.25; 0.01 and 0.005 μmol L^−^^1^ (Figure 5). The ciprofloxacin peak in each case considered was increasing along with the increase in t_acc_. Such behaviour on the eCNF/CNT/NiCo/GC electrode allows for optimal preconcentration times for different CIP concentrations, to increase the sensitivity of the measurements. Also, because of the adsorption-controlled mechanism of CIP oxidation, it was necessary to provide a stable and constant time of rest between each repetition of the CIP signal. In all the measurements considered, the rest time was set at 20 s.

### 3.5. Interferences

**Figure 6:**
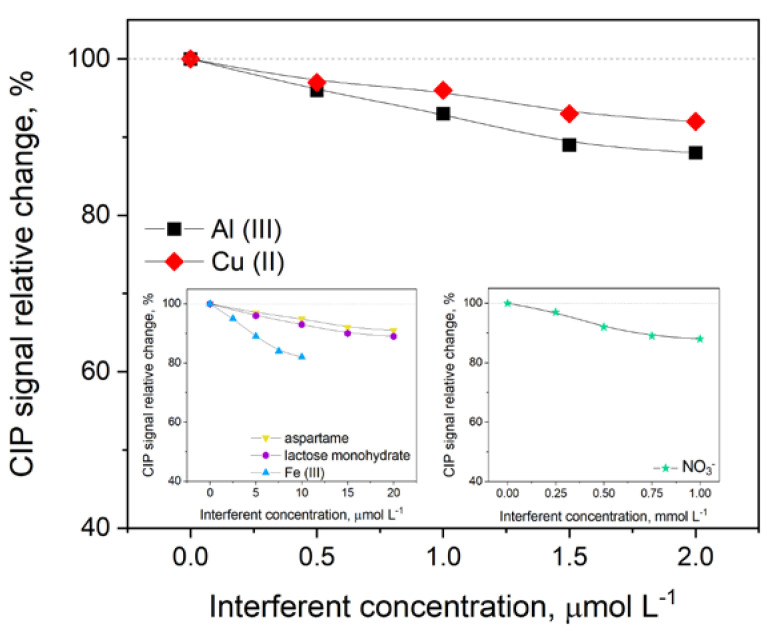
Dependence of the peak current on selected interferents with concentrations in the range from 0 to 1.0 mM for 0.5 µmol L^−1^ CIP concentration in 0.1 mol L^−1^ phosphate buffer (pH 6.8). Other instrumental parameters as in point 2.4.

The proposed voltametric method for ciprofloxacin determination was tested for the influence of different potential interferents on the CIP peak. The interferents may influence the analyte oxidation reaction in a few ways, resulting in the strengthening or suppression of its signal on the surface of the eCNF/CNT/NiCo/GC electrode. In this case, to the supporting electrolyte containing 0.5 µmol L^−^^1^ ciprofloxacin, organic and nonorganic potential interferents were added. Among all substances tested, the addition of Zn (II), Pb (II) (2 µmol L^−1^ each), Mn (II) (10 µmol L^−^^1^), CO_3_^2−^, NH_4_^−^, SO_4_^2−^ (1 mmol L^−^^1^), caffeine, microcrystalline cellulose, and magnesium stearate (20 µmol L^−1^) did not influence the peak of CIP. The manganese peak was registered during its addition to the supporting electrolyte but was separated and placed at a potential of 575 mV and did not have any influence on the CIP peak. Among the tested cations, Al (III), Cu (II) (2 µmol L^−1^ each), and Fe (III) (10 µmol L^−^^1^) were tested. Each of them caused a decrease in the CIP peak current of 12%, 8%, and 18%, respectively. Also, the addition of the nonorganic anion NO_3_^−^ in the amount of 1 mmol L^−^^1^ caused a decrease in the CIP peak of about 12%. Among the substances that can occur in tablet formulations as tablet fillers, aspartame and lactose monohydrate (20 µmol L^−1^) have a negative influence on the CIP peak, decreasing its height by about 9% and 11%, respectively. In Figure 6, the relative changes in the CIP signal caused by the addition of interference are presented.

### 3.6. Analytical Performance

**Figure 7:**
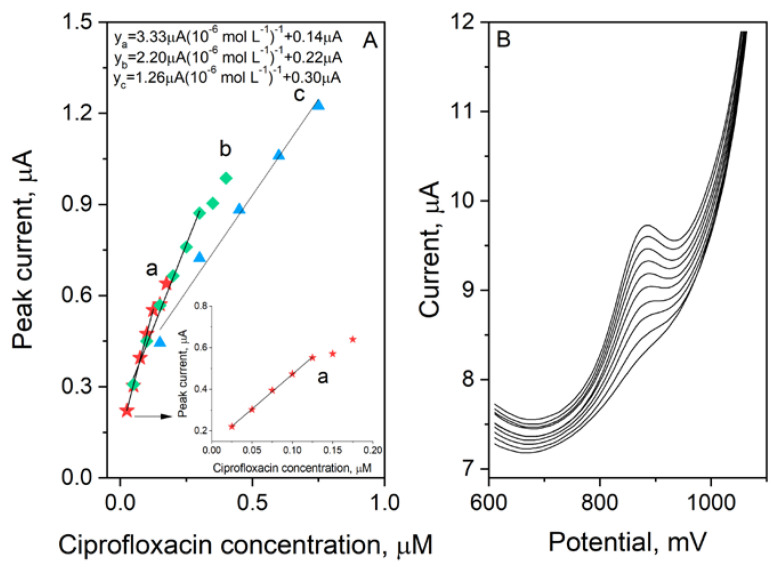
DPV ciprofloxacin calibration curves were registered for the preconcentration times (a) 45 s; (b) 30 s; (c) 15 s in 0.1 mol L^−1^ phosphate buffer (pH 6.8) (**A**) and corresponding voltammograms obtained for the preconcentration time of 30 s in the concentration range of 0.025–0.3 µmol L^−1^ (**B**). Other instrumental parameters are the same as in point 2.6.

Differential pulse voltammetry was applied for high-sensitivity CIP measurements both in the supporting electrolyte and in the complex matrices of real samples. DPV CIP voltammograms were recorded in the range from 0.025 to 0.3 µmol L^−1^ and the corresponding calibration curves are presented in Figure 7. In the mentioned range of concentrations, the linearity of the fitted regression line was very good, obtaining a value of R of about 0.998. The lowest detection limit (LOD) was calculated as 6.0 nmol L^−1^ with a sensitivity of 3.33 µA (µmol L^−^^1^)^−^^1^. The comparison of the LOD value of our method with other voltammetric methods described in the literature is presented in Table 1. The repeatability of the proposed sensor was calculated and expressed as RSD (relative standard deviation) using the voltammograms registered for the CIP concentration of 0.025 µmol L^−1^ and was equal to 2.4% (n = 7). The presented eCNF/CNT/NiCo/GC electrode has shown great stability of the CIP signal. It was possible to measure the CIP with unchanged sensitivity and signal height for a period of four weeks, which is equal to the registration of about 500 voltammograms.

To verify the proposed method, real samples were analysed for ciprofloxacin content. In the study, two types of antibiotic tablets from different producers containing 500 mg of CIP each, an antibiotic disc containing 5 µg of CIP each, and urine and plasma samples without the addition of CIP were used. The results of the analysis with the recovery values are presented in Table 2. The samples were prepared for analysis according to point 2.3.1. Plasma and urine samples were diluted 20 times prior to measurements. Taking into account the recovery values obtained in the range of 94–104%, it can be said that the proposed method of CIP determination in samples with complex matrices can be considered accurate.

## 4. Conclusions

In this paper, a highly sensitive adsorptive stripping voltammetric method for ciprofloxacine determination has been proposed. As the working electrode, a modification in the form of hierarchical electrospun carbon nanofibers with NiCo nanoparticles (eCNF/CNT/NiCo) on a glassy carbon electrode was used. The proposed sensor allows the CIP at concentrations as low as 6.0 nmol L^−^^1^ (LOD of the method). The described procedure was successfully applied for the determination of CIP in complex matrices, such as urine or plasma, and also in pharmaceutical products and antibiotic discs containing CIP with satisfactory recovery values, which ranged between 94–104%., which means that the CIP content in the tested samples was in good agreement with the producers’ declarations. The proposed electrode was characterised by great stability, with the possibility of use for about four weeks without any significant change in the CIP peak current, which was equal to the registration of about 500 voltammograms in this time, which is a great advantage of the proposed sensor. The repeatability of the CIP response on the eCNF/CNT/NiCo/GC is also very good; its value measured and expressed as RSD is equal to 2.4% for a CIP concentration of 0.025 µmol L^−1^ and seven registrations of the voltammogram. Also, the reproducibility of the CIP peak current measured on the five different modified electrodes was very good, at about 4.3%. The procedure for electrode preparation is quick and simple and does not involve the use of expensive apparatus. Therefore, considering the obtained results, it is possible to say that the developed method of ciprofloxacin determination on the eCNF/CNT/NiCo/GC electrode is a great alternative to the already presented assays.

## Figures and Tables

**Table 1:** Comparison of ciprofloxacin determination methods.

Electrode	Technique	Linear Range	LOD, mol L^−1^	References
ds-DNA-BDD	SWV	0.5–60 µM	0.44·10^−6^	[23]
rGO/PPR/GCE	DPV	0.002–400 µM	2.0·10^−9^	[31]
SUPRAS-AuNPs/AC/GCE	DPV	0.5–25 nM	0.20·10^−9^	[33]
PEI@Fe_3_O_4_@CNTS/GCE	DPV	0.03–70.0 µM	0.003·10^−6^	[34]
V_2_O_5_/SPE	DPV	0.04–365.0 µM	0.01·10^−6^	[49]
P-β-CD-L-arg/CPE	DPV	0.05–100 µM	0.01·10^−6^	[50]
CRGO/GCE	SWV	6–40 µM	0.21·10^−6^	[51]
MgFe_2_O_4_-MWCNTs	CV	0.10–1000 µM	0.01·10^−6^	[52]
MWCNT/GC	CV	3–1200 µM	0.9·10^−6^	[53]
Porous-Nafion-MWCNT/BDD	DPV	0.005–10 µM	0.005·10^−6^	[54]
PANI–β–CD/MWCNT/GCE	CV	10–80 µM	0.05·10^−6^	[55]
Ag-β-CD/GCE	DPV	0.1–50 nM	0.028·10^−9^	[56]
Cu-BTC	DPV	10 nM–20 µM	0.47·10^−9^	[57]
eCNF/CNT/NiCo/GC	DPV	0.025–0.3 µM	6.0·10^−9^	This work

SUPRAS-AuNPs/AC/GCE—gold nanoparticles deposited with waste coffee ground activated carbon on glassy carbon electrode combined with supra- molecular solvent; V_2_O_5_/SPE—screen-printed electrode modified with V_2_O_5_ nanoparticles; P-β-CD-L-arg/CPE—Poly-b-cyclodextrin-l-arginine carbon paste electrode; PEI@Fe_3_O_4_@CNTS/GCE—Polyethylenimine@Fe_3_O_4_@carbon nanotubes glassy carbon electrode; CRGO/GCE—Chemically reduced graphene oxide/glassy carbon electrode; MgFe_2_O_4_-MWCNTs—MgFe_2_O_4_-multiwall carbon nanotubes electrode; MWCNT/GC—multiwall carbon nanotubes modified glassy carbon electrode; Porous-Nafion-MWCNT/BDD—multiwalled carbon nanotubes dispersed in a porous Nafion film on a boron-doped diamond electrode; PANI–β–CD/MWCNT/GCE—glassy carbon electrode modified by a combination of multi-walled carbon nanotubes with β–cyclodextrin (β–CD) incorporated in a polyaniline film; Ag-β-CD/GCE—silver nanoparticle modified β-cyclodextrin glassy carbon electrode; Cu-BTC—Copper Based Organic Framework Modified Electrosensor; rGO/PPR/GCE—glassy carbon electrode modified with electro-polymerized phenol red and reduced graphene oxide; ds-DNA-BDD—boron doped diamond electrode modified with double-stranded dsDNA; eCNF/CNT/NiCo/GC—glassy carbon electrode modified with hierarchical nanocomposite—electrospun carbon nanofibers/carbon nanotubes/NiCo nanoparticles.

**Table 2:** Results of ciprofloxacin determination in different samples.

Sample	Added, mg	Found, mg	Recovery, %
Tablet 1	0	521	104
	500	1028	103
	1000	1508	101
	1500	1973	98
Tablet 2	0	513	103
	500	1024	104
	1000	1535	103
	1500	1969	98
Producer declares 500 mg ciprofloxacin per tablet			
**Sample**	**Added, μg**	**Found, μg**	**Recovery, %**
Antibiotic disc	0	5.12	102
	5	10.04	101
	10	14.92	99
Producer declares 5 μg ciprofloxacin per disc			
**Sample**	**Added, μmol L^−1^**	**Found, μmol L^−1^**	**Recovery, %**
Urine (diluted 20×)	0	ND	-
	2	1.88	94
	4	4.04	101
	6	6.26	104
	8	7.80	97
Plasma (diluted 20×)	0	ND	-
	0.5	0.47	94
	1.0	1.04	104
	1.5	1.48	99
	2.0	1.93	97
ND—not detected			

## Data Availability

The data presented in this study are available on request from the corresponding author.
